# Analysis of HFE gene mutations and HLA-A alleles in Brazilian patients with iron overload

**DOI:** 10.1590/S1516-31802006000200002

**Published:** 2006-03-02

**Authors:** Rodolfo Delfini Cançado, Aline Cristiane de Oliveira Guglielmi, Carmen Silvia Vieitas Vergueiro, Ernani Geraldo Rolim, Maria Stella Figueiredo, Carlos Sérgio Chiattone

**Keywords:** Ferritin, Iron overload, Hemochromatosis, Iron metabolism disorders, Inborn genetic diseases, Ferritina, Sobrecarga de ferro, Hemocromatose, Doenças do metabolismo do ferro, Doenças genéticas inatas

## Abstract

**CONTEXT AND OBJECTIVE:**

Hemochromatosis is a common inherited disorder of iron metabolism and one of the most important causes of iron overload. The objective was to analyze the presence of C282Y, H63D and S65C mutations in the HFE gene and HLA-A alleles for a group of Brazilian patients with iron overload, and to correlate genotype with clinical and laboratory variables.

**DESIGN AND SETTING:**

Prospective study, in Discipline of Hematology and Oncology, Faculdade de Ciências Médicas da Santa Casa de Misericórdia de São Paulo.

**METHODS:**

We studied 35 patients with iron overload seen at our outpatient unit between January 2001 and December 2003. Fasting levels of serum iron and ferritin, and total iron- binding capacity, were assayed using standard techniques. Determinations of C282Y, H63D and S65C mutations in the HFE gene and of HLA-A alleles were performed by polymerase chain reaction (PCR).

**RESULTS:**

Twenty-six out of 35 patients (74%) presented at least one of the HFE gene mutations analyzed. Among these, five (14%) were C282Y/C282Y, four (11%) C282Y/H63D, one (3%) H63D/H63D, six (17%) C282Y/WT and ten (29%) H63D/WT. No patients had the S65C mutation and nine (25%) did not present any of the three HFE mutations. Four out of five patients with C282Y/C282Y genotype (80%) and three out of four patients with C282Y/H63D genotype (75%) were HLA A*03.

**CONCLUSION:**

Analysis of HFE gene mutations constitutes an important procedure in identifying patients with hereditary hemochromatosis, particularly for patients with iron overload.

## RESUMO

**CONTEXTO E OBJETIVO:**

Hemocromatose é um distúrbio hereditário comum do metabolismo do ferro e uma das causas mais importantes de sobrecarga de ferro. O objetivo foi analisar a presença das mutações C282Y, H63D e S65C no gene HFE e dos alelos HLA-A em um grupo de pacientes brasileiros com sobrecarga de ferro e correlacionar o genótipo com variáveis clínicas e laboratoriais.

**TIPO DE ESTUDO E LOCAL:**

Estudo prospectivo, na Disciplina de Hematologia e Oncologia. Faculdade de Ciências Médicas da Santa Casa de Misericórdia de São Paulo.

**MÉTODOS:**

Estudamos 35 pacientes com sobrecarga de ferro atendidos em nosso ambulatório entre janeiro de 2001 e dezembro de 2003. Ferro sérico, ferritina sérica e capacidade total de ligação de ferro foram determinados por técnicas convencionais. As mutações C282Y, H63D e S65C do gene HFE e a determinação dos alelos HLA-A foram realizadas por reação de polimerase em cadeia (PCR).

**RESULTADOS:**

Vinte e seis dos 35 pacientes (74%) apresentavam pelo menos uma das mutações analisadas do gene HFE. Entre esses, cinco (14%) com genótipo C282Y/C282Y, 4 (11%) C282Y/H63D, 1 (3%) H63D/H63D, 6 (17%) C282Y/WT e 10 (29%) H63D/WT. Não foi encontrado nenhum paciente com a mutação S65C e 9 (26%) pacientes não apresentavam nenhuma das três mutações do gene HFE. Quatro dos 5 pacientes com genótipo C282Y/C282Y (80%) e 3 dos 4 pacientes C282Y/H63D (75%) eram HLA A*03.

**CONCLUSÃO:**

Análise das mutações do gene HFE constitui um importante procedimento na identificação de pacientes com hemocromatose hereditária, particularmente em pacientes com sobrecarga de ferro.

## INTRODUCTION

Iron homeostasis is maintained by regulating iron absorption. Unlike for other essential minerals, the human body does not have a regulatory mechanism for excreting excess iron. Therefore, although iron uptake can be stimulated when necessary, its absorption must be otherwiselimited, so as to prevent toxicity due to iron overload. Iron deficiency remains the most important micronutrient deficiency worldwide, but increasing awareness of the genetic basis for iron loading diseases points towards iron overload as amajor public health issue as well.^1,2^

The normal quantity of total body iron in adult individuals usually ranges from 50 to 60 mg/kg of body weight in males and 35 to 40 mg/kg in females. Iron overload results from many disorders and may lead to the development of organ damage and increased mortality, especially when the total body iron stores are more than five grams.^2^

Iron overload is a frequent metabolic abnormality. It may be either primary, resulting from lack of regulation of intestinal iron absorption, as in genetic or hereditary hemochromatosis, or secondary. In the case of secondary overload, it may be due to or associated with one of the following: ineffective erythropoiesis (as in β-thalassemia, sideroblastic anemia or aplastic anemia, for example), in which there is both an increase in iron absorption and frequently a need for repeated transfusions; chronic liver disease (e.g. alcoholic cirrhosis, viral hepatitis, etc.); or ingestion of excessive amounts of iron.^3,4^

Hereditary hemochromatosis (HH) is a common autosomal recessive disorder of iron metabolism in Caucasians, with a prevalence of one in 300-500 individuals. This disease is characterized by enhanced gastrointestinal absorption of dietary iron that leads to progressively increased body iron stores and multiple organ dysfunction. If it remains undiagnosed and untreated by phlebotomy, severe clinical complications may occur in the fifth or sixth decade of life. While some of these complications, such as cirrhosis (and increased susceptibility to hepatocarcinoma), diabetes and cardiac failureor arrhythmia, definitively affect patient survival, others, such as endocrine complications (hypogonadism) and arthritis, markedly reduce the quality of life of HH patients. Early diagnosis and treatment, however, can completely prevent the development of clinical complications and offers patients a normal life expectancy.^1-3,5-7^

Diagnosis is dependent onphenotypic expression, but this may be modifiedby non-ge- netic factors such as age, dietary composition, blood donation, menstruation, pregnancy, pathological blood loss and coexistence of other diseases that affect iron metabolism, e.g. heterozygous β-thalassemia, hepatitis, hereditary spherocytosis, etc.^5^

Investigation of the HH gene began 20 years ago, when its genetic localization close to the major histocompatibility complex (MHC) was established fairly accurately. This was based on linkage analysis in families with several siblings affected, mostly presenting histocompatibility leukocyte antigen (HLA) serotype A*03. Considering that genetic studies indicated that the HH gene was very close to HLA-A, a major effort was made to search for the gene in the region surrounding HLA-A.^8,9^

In 1996 a gene involved in HH pathogenesis, called HFE, was identified on the short arm of chromosome 6. The gene encodes an HLA-A class 1-like protein involved in the regulation of cellular iron metabolism.^1,10,11^

Three allelic variants of HFE have been correlated with HH. In approximately 60% to 90% of the cases of HH, the defect is a single missense mutation at position 282, where cysteine is replaced by tyrosine (C282Y). The C282Y mutant HFE protein is unable to bind to β_2_ -microglobulin, which results in unregulated transferrin receptor-mediated iron uptake in the gut.^5,8,12^

A second mutation has been found at position 63, where histidine is replaced by aspartate (H63D). The H63D mutation, while able to bind to transferrin receptors, appears to lack the normal high degree of inhibitory effect on the transferrin receptor. Individuals who are homozygous for the H63D mutation (H63D/H63D) and those who are compound heterozygous (C282Y/H63D) have a low rate of phenotypic expression. These account for approximately 5% and 15% of the cases of hereditary hemochromatosis, respectively.^13-18^

A third mutation, which results in the substitution of cysteine for serine at amino acid position 65 (S65C), has an estimated heterozygote frequency of 4%. This may be implicated in iron storage disease, usually in the compound heterozygous state with C282Y (C282Y/S65C).^19,20^

The prevalence of the C282Y mutation among healthy Brazilian subjects (carrier frequency: 4.4%) is lower than what is observed among Europeans. These findings can be explained by the different ethnic compositions of these populations.^21,22^ The H63D mutation has been found to be highly prevalent among Brazilians (carrier frequency: 27.5%), with a frequency similar to what is observed among white Europeans, particularly among Italians.^22^

## OBJECTIVE

The aims of the present study were to determine the prevalence of C282Y, H63D and S65C mutations in the HFE gene and HLA-A alleles in a selected group of Brazilian patients with iron overload, and to correlate genotypes with clinical and laboratory variables.

## METHODS

Between January 2001 and December 2003, 35 patients were referred by general practitioners and visited our unit for investigation of iron overload disease. The diagnosis of iron overload was confirmed by two fasting measurements of the transferrin saturation index (TS), with results ≥ 60% in males and ≥ 50% in females, and/or serum ferritin concentrations exceeding 300 ng/ml in males or 200 ng/ml in females.

Informed consent was obtained from all subjects and the study protocol was approved by the Ethics Committee of our institution.

### Clinical evaluation

The following clinical data were recorded for all subjects, using the same questionnaire: age; gender; any awareness of a family relative regularly phlebotomized due to iron excess; any personal history of blood donation; parenteral or dietary iron overload; blood transfusions; number of pregnancies; use of oral contraception and intrauterine device; menstrual history; any history of excessive alcohol consumption (> 60 g/day) and chronic liver disease due to hepatitis B and C; non-alcoholic steatohepatitis and cirrhosis; chronic fatigue, chronic distal arthralgia, impotence, diabetes mellitus and chronic hemolytic anemia.

### Laboratory evaluation

Fasting levels of serum iron (normal: 60-160 µg/dl in men; 40-145 µg/dl in women) and serum ferritin (normal: 30-300 µg/l in men and postmenopausal women; 8-250 µg/l in premenopausal women; Baxter kit) were determined by the enzyme immunoassay method. Total iron-binding capacity (TIBC) was assayed using standard techniques. The transferrin saturation index was calculated using the formula: [serum iron/TIBC] x 100 (normal: 20-45%). Alanine aminotransferase, gammaglutamyl transpeptidase, glucose, cholesterol, triglycerides, hemoglobin electrophoresis, lactate dehydrogenase, reticulocyte count, red blood cell count and serum assays for hepatitis B and C were also determined for all subjects.

### HFE gene mutation analysis

Genomic DNA was extracted from peripheral blood leukocytes using the fresh and cryopreserved clotted human blood method described by Salazar et al. (1998).^23^ HFE genotyping for the C282Y, H63D and S65C mutations was performed by the polymerase chain reaction (PCR) to amplify the genomic samples of DNA taken from each patient. The productswere digested by adding a restriction enzyme, and analyzed bymeans of electrophoresis on 2% agarosegels.

The primer sequences used for identification of the C282Y mutation were: 5' – TGC CTC CTT TGG TGA AGG TGA CAC – 3' and 5' – CTC AGG CAC TCC TCT CAA CC – 3'. For the identification of the H63D and S65C HFE gene mutations, the following primers were used: 5'- TCA CAC TCT CTG CAC TAC CTC TTC ATG G – 3' and 5'– TAC ACA GTG AAC ATG TGA TCC CAC C – 3'. *Rsa*I (C282Y analysis), Dpn II (H63D analysis) and Hinf I (S65C analysis) restriction enzyme digestion were utilized following PCR genomic amplification, to determine the HFE genotypes. This was performed in accordance with the methods described by Feder et al. (1996)^11^ and Simonsen et al. (1999).^24^

### HLA typing

Determination of HLA-A alleles was performed using One Lambda kits (One Lambda, California, United States).

### Statistical analysis

Differences between the transferrin saturation index and ferritin concentration, according to HFE genotype, were evaluated by means of the non-parametric Mann-Whitney test and Kruskal-Wallis test; p values of less than 0.05 were considered statistically significant.

All data entries and calculations were performed using the Excel software (Microsoft Corp.).

## RESULTS

Twenty-six (74%) out of the 35 patients with iron overload presented at least one of the HFE gene mutations analyzed, as can be seen in Table 1. No patient studied was found to have the S65C mutation and nine (25%) did not present any of the three HFE mutations.

**Table 1:** Distribution of the 35 Brazilian patients with iron overload according to the HFE genotype

Genotype	n	%
C282Y/C282Y	5	14
C282Y/H63D	4	11
H63D/H63D	1	3
C282Y/WT	6	17
H63D/WT	10	29
WT/WT	9	26
**Total**	**35**	**100**

* *n = number of subjects tested; WT = wild type.*

In 16 (46%) out of the 35 patients, iron overload was associated with another disease or factor related to iron accumulation. The distribution of the patients according to genotype, and the presence or absence of secondary factors associated with iron overload, is shown in Table 2.

**Table 2:** Distribution of the 35 Brazilian patients according to the genotype and the presence or absence of other disease or factor associated with iron overload

Genotype	Anti-HCV positivity	Excessive alcohol consumption	Chronic hemolytic anemia	Without secondary factor identified
	n (%)	n (%)	n (%)	n (%)
C282Y/C282Y (n = 5)	0 (0%)	0 (0%)	1 (25%)	4 (75%)
C282Y/H63D (n = 4)	0 (0%)	0 (0%)	2 (50%)	2 (50%)
H63D/H63D (n = 1)	0 (0%)	1 (100%)	0 (0%)	0 (0%)
C282Y/WT (n = 6)	1 (17%)	2 (33%)	1 (17%)	2 (33%)
H63D/WT (n = 10)	1 (10%)	0 (0%)	2 (20%)	7 (70%)
WT/WT (n = 9)	1 (10%)	2 (22,5%)	2 (22,5%)	4 (45%)
**Total (n = 35)**	**3 (9%)**	**5 (14%)**	**8 (23%)**	**19 (54%)**

*HCV = hepatitis C virus.*

The following distribution was observed among the chronic hemolytic anemia patients: one with β-thalassemia trait (H63D/WT); three with ∝-thalassemia (two C282Y/H63D and one H63D/WT); two with hereditary spherocytosis (one C282Y/C282Y and one C282Y/WT) and two patients with sickle cell anemia. Among this group of patients, only the sickle cell patients had received blood transfusions. One 28-year-old patient had received 10 units so far and the other 63-year-old patient had received four units of blood transfusions. Both of them were female and presented WT/WT genotype.

Twenty-two (63%) patients were male and 13 (37%) female; their median age was 50 years (range: 25-78 years); 26 (80%) of these patients were between 40 and 79 years old; and 29 (83%) were white. The main clinical characteristics and iron status of the 35 patients according to the HFE genotype are shown in Table 3.

**Table 3:** Main clinical characteristics and iron status of the 35 Brazilian patients with iron overload according to the HFE genotype

	Genotype					
	C282Y/C282Y (n = 5)	C282Y/H63D (n = 4)	H63D/H63D (n = 1)	C282Y/WT (n = 6)	H63D/WT (n = 10)	WT/WT (n = 9)
**Age (years)**						
Median	57	43.5	44	55.5	48.5	51
Min - max	41-67	25-63	--	44-67	40-71	28-78
**Sex**						
Male n = 22 (63%)	2	2	1	4	7	6
Female n = 13 (37%)	3	2	0	2	3	3
**Ethnicity**						
White n = 29 (83%)	5	4	1	5	8	6
Non-white n = 9 (17%)	0	0	0	1	2	3
**Transferrin saturation index (%)**						
Mean (SD)	77.50* (9.57)	63.75 (6.18)	77	58.50 (12.47)	61.15 15.50)	53.54 29.25)
Min - max	62.50-87	57-69	--	36-69	32.50-92	20.30-90
**Serum ferritin concentration (ng/ml)**						
Mean (SD)	2,264.80† (1714)	1,259.50 (927)	1200	1,413.83 (500)	1,139 (721)	1,648.33 (1692)
Min - max	1,000-5,265	537-2,620	--	890-2,324	500-2,500	696-5,913

*SD = standard deviation; min = minimum; max = maximum; n = number of subjects in each group;*

* *= p < 0.01, for C282Y/C282Y versus other genotypes;*

† *= p < 0.001, for C282Y/C282Y versus other genotypes.*

Table 4 shows the HLA-A frequency determined for 21 patients according to the HFE genotype.

**Table 4:** Determination of HLA-A frequency according to the HFE genotype for 21 patients with iron overload in Brazil

HLA	Genotype					
	C282Y/C282Y (n = 5)	C282Y/H63D (n = 4)	H63D/H63D (n = 1)	C282Y/WT (n = 6)*	H63D/WT (n = 10)†	WT/WT (n = 9)‡
A*03 positive	4	3	0	–	1	1
A*03 negative	1	1	1	1	3	5
**Total**	5	4	1	1	4	6

* *Test performed on only one patient;*

† *test performed on four patients;*

‡ *test performed on six patients; HLA = histocompatibility leukocyte antigen.*

## DISCUSSION

Iron overload can be associated with various pathological conditions.^25^ We studied 35 patients who were referred by general practitioners and visited our unit for investigation of iron overload disease. It is important to emphasize that this study is not applicable to the general population, because the focus was on preselected patients sent for hematological consultation.

Although hemochromatosis is the most common cause of primary iron overload, there are other inheritable causes of primary iron overload and many secondary congenital or acquired pathological conditions that are related to iron accumulation.^2,3,5,25,26^

The median age of the patients homozygous for C282Y was 57 years (range: 41-67 years), whereas for the patients with other genotypes the median age ranged from 43.5 years to 55.5 years.

In spite of improvements over recent years, the diagnosis of HH is still often missed or delayed. This can be attributed to lack of recognition of the disease, confusion regarding symptoms that are also present in other diseases, and incomplete phenotypic expression in some affected individuals. These factors may explain why the homozygous patients showed higher median age than did the patients with other genotypes, in addition to the small number of patients enrolled in this study.

Although all these patients had iron overload, the individuals homozygous for C282Y had significantly higher transferrin saturation and ferritin levels (77.5% and 2264.80 ng/ml, respectively) than did those with other mutations (p < 0.001). In four out of five patients (80%), the C282Y/C282Y iron overload was not associated with any other factor or disease, whereas one patient (20%) was found to have hereditary spherocytosis.

The presence of the HFE mutations known to cause the disease (C282Y/C282Y, C282Y/H63D and possibly H63D/H63D), and direct evidence (raised hepatic iron concentration) or indirect evidence (raised transferrin saturation or ferritin level) of increased iron stores, constitute the current gold standard for definitive diagnosis of he- mochromatosis.^2,5,10^

Genotyping patients for C282Y and H63D mutations is useful for confirming the diagnosis of HH and for family studies. Most European studies have reported that 60-90% of typical hemochromatosis patients are homozygous for the C282Y mutation of the HFE gene (C282Y/C282Y). Compound heterozygotes (C282Y/H63D) and, less commonly, H63D homozygotes usually have normal iron tests, but it has been described that in some cases these genotypes may resemble C282Y homozygotes, with mild-to-moderate iron overload.^4,5,9,15^

In the present study, 26 out of the 35 patients (74%) presented at least one of the HFE gene mutations. Among these, five (14%) were C282Y/C282Y, four (11%) C282Y/H63D, one (3%) H63D/H63D, six (17%) C282Y/WT and ten (29%) H63D/WT. No patient studied was found to have the S65C mutation and nine (25%) did not present any of the three HFE mutations. Thus, we confirmed the diagnosis of HH in ten (28%) patients.

Altes et al. (2003) observed that HH was the most frequent cause of iron overload: 44/150 patients (29%). Among these patients, 24 were C282Y/C282Y, 14 C282Y/H63D and six H63D/H63D.^27^ Bittencourt et al. (2002) showed the presence of the C282Y/ C282Y genotype in roughly half of their Brazilian patients with HH.^28^

The clinical expression of HH occurs mostly in homozygotes. Heterozygotes may have minor abnormalities of iron status parameters. However, it has been observed that patients with single HFE gene mutations may have clinical manifestations and may develop significant iron overload when other diseases or environmental factors coexist. Under these conditions, relatively mild-to-moderate iron overload has been observed, resulting in a hemochromatosis-like phenotype.^2,3,5,29-31^

Our results support this hypothesis. We found sixteen patients (46%) heterozygous for C282Y or H63D. In seven (44%) of these patients, iron overload was associated with anti-HCV positivity (two cases), alcoholism (two cases) and chronic hemolytic anemia (three cases). Accordingly, Alter et al. (2003) observed that the hepatitis C virus was the second biggest cause of iron overload: 33/150 patients (22%). Sixteen of these 33 patients (48%) were H63D/WT.^27^

Erhardt et al. (2003) demonstrated that C282Y and H63D heterozygosity are independent risk factors for liver fibrosis and cirrhosis in patients with chronic hepatitis C. They suggested that screening for HFE mutations should be considered in cases of HCV infection.^32^Tung et al. (2003) showed that the presence of HFE mutations is independently associated with iron loading and advanced fibrosis among patients with compensated liver disease from chronic hepatitis C. Their results suggested that HFE mutations accelerate hepatic fibrosis in hepatitis C, but may not be responsible for progression to end-stage liver disease.^33^

It is important to recognize that there are many causes of iron overload other than hemochromatosis. Some patients have a clinical condition indistinguishable from that of genetic hemochromatosis but do not present any mutation of the HFE gene.

Nine out of our 35 patients (25%) did not present any of the three HFE mutations analyzed. Among these, five (55%) presented another disease or factor related to iron accumulation: chronic hemolytic anemia (two cases), alcoholism (two cases) and anti-HCV positivity (one case).

Pietrangelo (2003)^2^ and Altes et al. (2003)^27^ observed that HH can occur in adults who do not have any mutation of the HFE gene. This suggests the existence of one or more distinct genetic diseases that may cause a type of adult hereditary iron overload other than that associated with the HFE gene.^2,27^

An increasing number of mutations in other genes (ferroportin, transferrin receptor 2 and aceruloplasminemia) have been identified as causing iron overload. There will likely be additional hemochromatosis mutations found in the future. If iron overload is present without any mutations in the HFE gene, a careful review of the history for other risk factors must be made. Liver biopsy may be useful in determining the cause of the iron overload and the need for treatment.^34-36^

After identifying the HFE gene and its different mutations, genotyping diagnosis becomes a simple and important tool in the diagnostic work-up for iron overload patients. However, before this became possible, the gene could be identified indirectly through HLA-linked genes. In this study, the frequency of HLA A*03 in patients with the C282Y/C282Y genotype was 70%, while the frequency for the non-homozygous C282Y subgroup was 20-30%. This was the same as the frequency observed in the general population.

Despite the small number of patients enrolled in the present study, the results suggest that HLA A*03 is clearly associated with the C282Y/C282Y and C282Y/H63D genotypes.

## CONCLUSION

Analysis of HFE gene mutations is an important procedure for identifying patients with hereditary hemochromatosis, particularly in cases of iron overload.

Heterozygosity for C282Y or H63D can be associated with significant iron overload, particularly when associated with other factors or diseases related to iron accumulation, such as chronic hemolytic anemia, hepatitis C and alcoholism.

The presence of iron overload in patients who do not have HFE gene mutations supports the evidence that genes other than HFE may be involved in iron loading.
